# Benign Pneumatosis Intestinalis in a Patient With Symptomatic COVID-19

**DOI:** 10.7759/cureus.78604

**Published:** 2025-02-06

**Authors:** Sun Yu, Adeline Fleitz, Artem Shmelev

**Affiliations:** 1 Surgery, Stony Brook University, Stony Brook, USA

**Keywords:** benign pneumatosis intestinalis, coronovirus, covid-19, pneumatosis intestinalis, sars-cov-2

## Abstract

The pathophysiology of pneumatosis intestinalis (PI) is poorly understood. PI can be associated with COVID-19 infection. Although this relationship is unclear, proposed mechanisms include direct viral invasion of the mucosa, the use of IL-6 inhibitors, and bowel ischemia. We present a case of a 75-year-old female with benign PI after symptomatic COVID-19 following recovery from a recent abdominal operation. The patient presented with obstructive symptoms, dyspnea, and tachycardia. Workup revealed PI with leukocytosis and lactic acidosis. She was taken emergently to the operating room for exploration, which revealed a short segment of otherwise healthy jejunum with PI. She recovered well and was discharged back to a facility. This case differs by the benign nature of PI and the possible direct effect of COVID-19 on the small bowel. Benign PI in the setting of COVID-19 infection may be secondary to the disease process itself rather than its treatment modality or hypercoagulability. This potential association needs to be considered when evaluating the patient with incidental PI on imaging. Patients with chronic constipation and underlying lung disease may be at a higher risk of developing PI, with varying clinical significance.

## Introduction

Pneumatosis intestinalis (PI) is defined as a radiologic demonstration of gas within the wall of the small or large bowel, the etiology of which can herald a benign or life-threatening disease course [1]. Accompanying portal or portomesenteric venous gas is a more ominous sign, indicating likely transmural bowel necrosis [2]. With variable etiologies, the pathophysiology of PI is poorly understood. Suggested mechanisms include mechanical, pulmonary, and bacterial theories [3]. The mechanical theory states that the intraluminal bowel gas enters the wall through a mucosal defect or through the serosa via the mesenteric vessels. The pulmonary theory implicates alveolar rupture due to lung disease as a cause of gas introduction into the mesenteric vasculature. In the bacterial theory, the invasion of the intraluminal compartment by gas-producing bacterial species is to blame.

A wide range of secondary causes of PI has been described in the literature, including chronic obstructive pulmonary disease (COPD), ischemic, necrotic, and obstructive insults, as well as celiac disease, amyloidosis, AIDS, and drugs including chemotherapies, steroids, and immunomodulating agents [1].

Since the start of the global COVID-19 pandemic, multiple cases of PI in the setting of COVID-19 infection have been documented in the literature [4-7]. One case demonstrated a patient with PI and pneumoperitoneum as the sole manifestation of their COVID-19 infection, resolved with conservative management [8]. A case series of nine patients with PI in the setting of COVID-19 demonstrated this radiological sign as a poor prognostic factor with 77.8% mortality and an average time to death of less than a week from the time of PI discovery. Of note, most of these patients had lactic acidosis with the average lactate at the time of diagnosis of PI at 4.33 mmol/L in this patient population [9].

Pathophysiologic mechanisms for severe acute respiratory syndrome coronavirus 2 (SARS-CoV-2) causing PI have been proposed to involve the use of the angiotensin-converting enzyme 2 (ACE2) receptors highly expressed in the epithelial cells of the gastrointestinal tract and liver [10]. Using ACE2 receptors to enter the cells, SARS-CoV-2 may be able to invade the cells of the gastrointestinal tract; however, there has not been an association between fecal viral load and gastrointestinal manifestations [10]. The prevalence of GI symptoms in COVID-19 infection is estimated to be roughly 18%, including anorexia, nausea, vomiting, diarrhea, and abdominal pain [11]. Up to 16% of patients with COVID-19 present solely with gastrointestinal manifestations, often being diagnosed incidentally through imaging with small and large bowel wall thickening, fluid-filled colon, PI, pneumoperitoneum, intussusception, and ascites as the most common findings [11]. One single-institution study of a small cohort of patients who underwent gastrointestinal resections with concomitant active COVID-19 infection found pneumatosis cystoides intestinalis in all cases, entailing cyst formation with multinucleated giant cell aggregates within prominent submucosal edema. Other histological findings varied extensively from acute superficial colitis to frank necrosis with microthrombi. Many of these patients were acutely ill, demonstrating ischemic enterocolitis, with three of four succumbing to their disease on the same admission [12].

Here we present a case of benign PI in a patient with COVID-19 infection presenting with sepsis and clinical and radiologic suspicion for small bowel perforation.

## Case presentation

The patient is a 75-year-old female with a past medical history of hypertension, hyperlipidemia, schizophrenia, and seizure disorder requiring multiple antipsychotic and antiseizure medications, chronic constipation with a history of bowel obstruction requiring laparotomy and small bowel resection (SBR) years ago, and right breast cancer status-post simple mastectomy. She presented with a two-day history of abdominal pain, nausea, emesis, and obstipation, as well as shortness of breath and tachycardia for one day. Notably, the patient was admitted to our institution one month prior for an internal hernia due to a mesenteric defect from remote SBR, causing closed-loop small bowel obstruction and necrosis. This required exploratory laparotomy with another SBR. She fully recovered from this surgery, regained normal bowel function, and was discharged to a rehabilitation facility. There the patient contracted a symptomatic COVID infection, which was diagnosed 10 days prior to the current presentation and was clinically improving after five days of molnupiravir.

In the ED, the patient was tachycardic in the low 100s and required supplemental oxygen for oxygen saturation in the 80s. She had a mild productive cough and a distended abdomen, uncomfortable to palpation. Relevant laboratory data are listed in Table 1.

**Table 1:** Relevant laboratory data on presentation SARS CoV-2 PCR: severe acute respiratory syndrome coronavirus 2 polymerase chain reaction

Laboratory measure	Value	Reference range
Serum lactate	3.6 mmol/L	0.5-2.0 mmol/L
Serum bicarbonate	26 mmol/L	21-31 mmol/L
Anion gap	14 mmol/L	9-16 mmol/L
WBC count	13.9 K/µL	4.8-10.8 K/µL
SARS CoV-2 PCR (nasopharynx)	Positive	Negative

**Figure 1:**
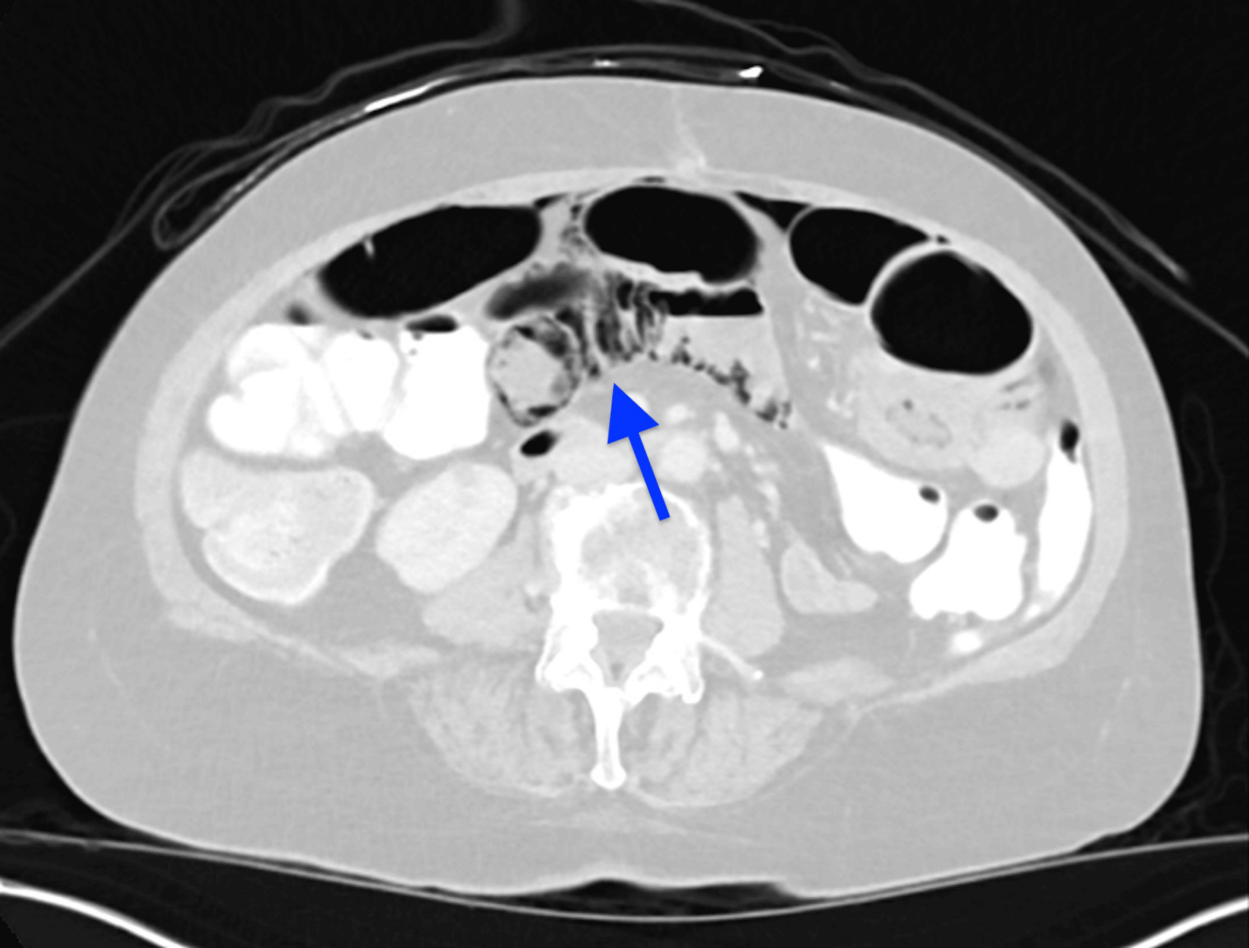
Representative image from the CT scan of the abdomen showing extensive small bowel pneumatosis (blue arrow)

**Figure 2:**
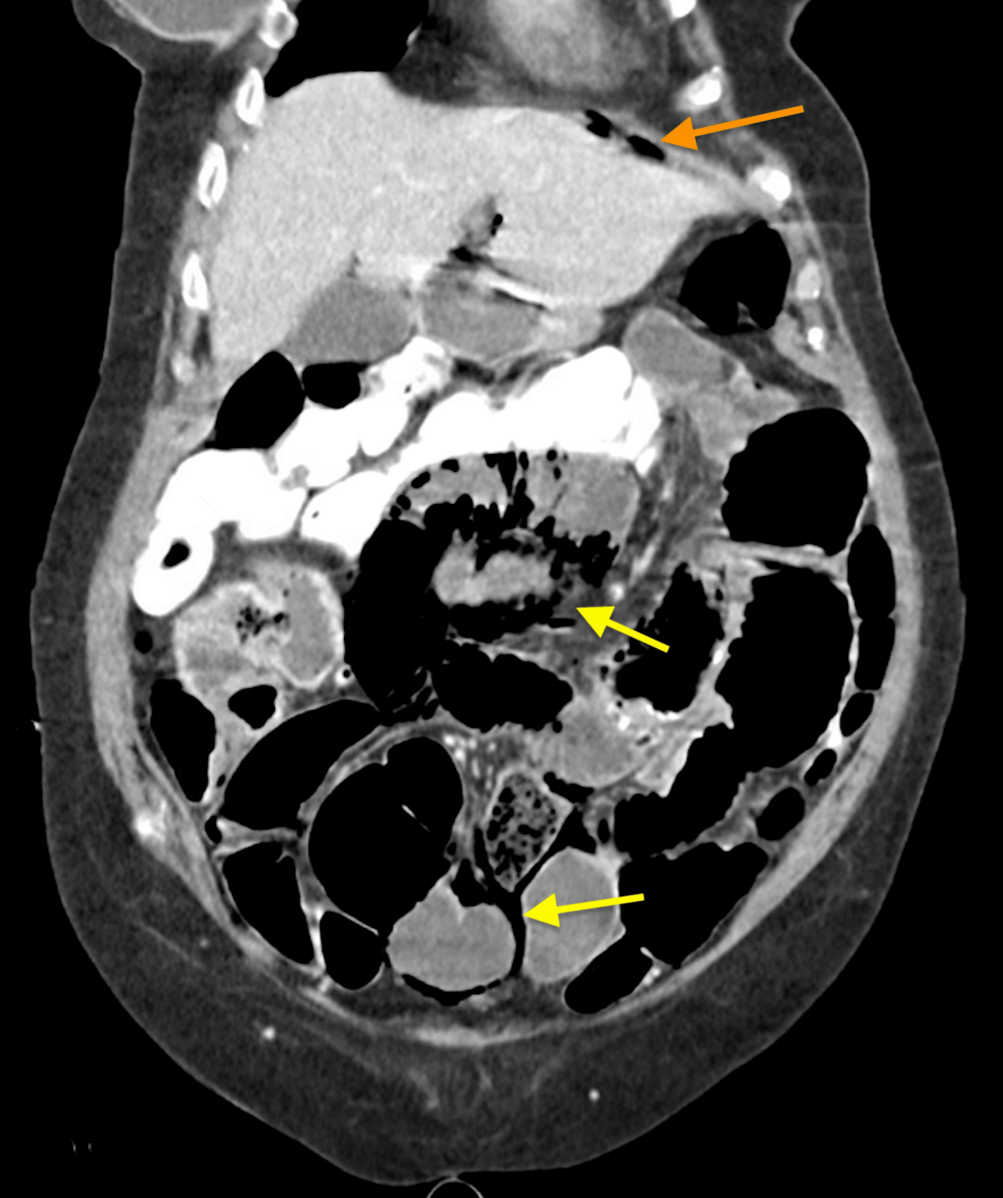
Small volume pneumoperitoneum (orange arrow) noted with extensive pneumatosis intestinalis (yellow arrows). Enteric contrast from three days prior for assessment of constipation noted in the colon

**Figure 3:**
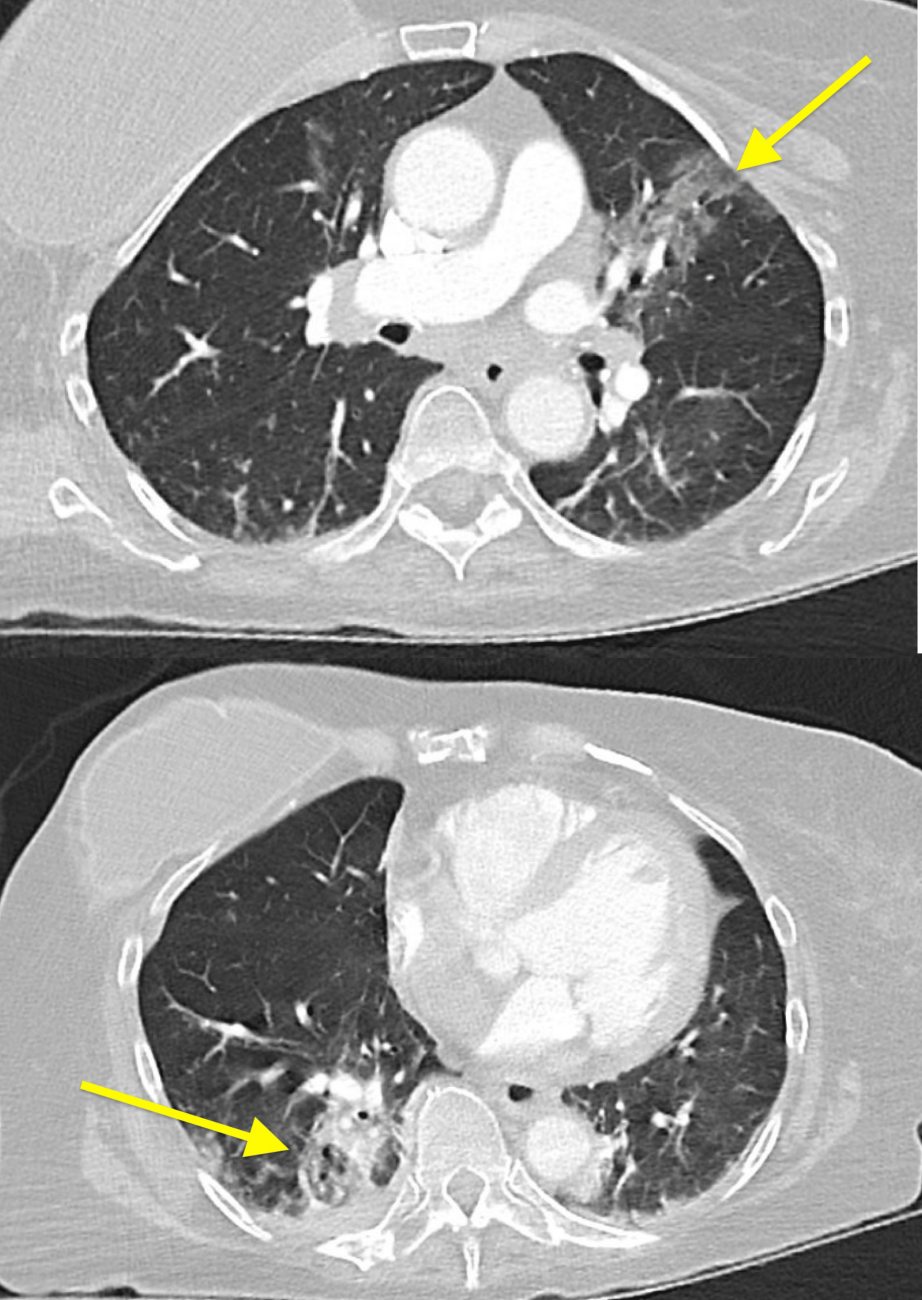
Left upper and right lower lobes consolidation (yellow arrows)

CT scan demonstrated extensive pneumatosis of multiple small bowel loops with small volume pneumoperitoneum concerning small bowel perforation (Figures 1-2). CT pulmonary angiography was negative for pulmonary embolism but showed left upper and right lower lobe consolidation concerning pneumonia (Figure 3).

**Figure 4:**
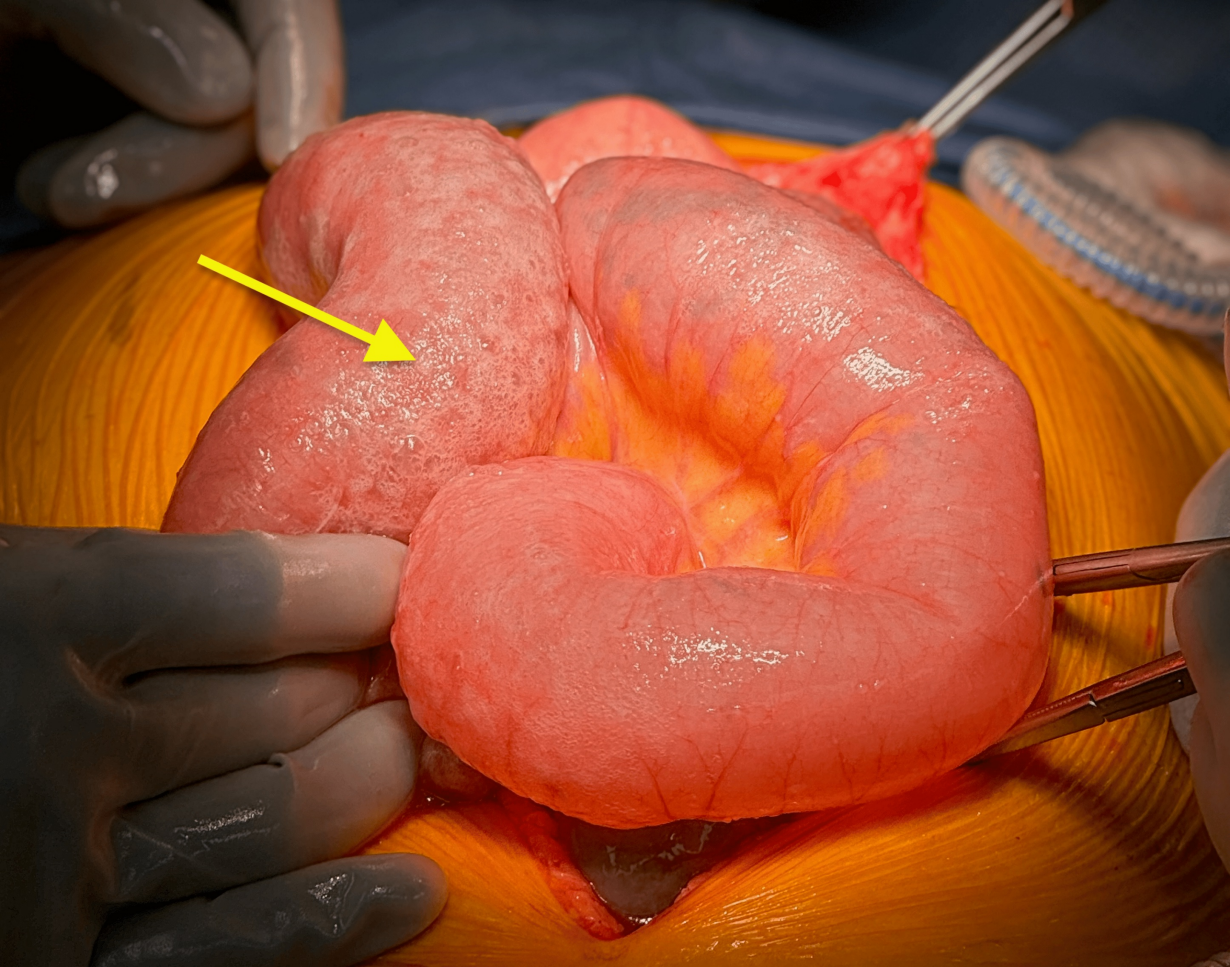
Intraoperative photograph of the diffuse pneumatosis of otherwise healthy jejunum (yellow arrow)

Given the recent surgical history, symptoms of bowel obstruction with abdominal pain and distension, lactic acidosis, leukocytosis, and imaging findings concerning small bowel perforation with PI, the patient was taken emergently to the operating room for exploratory laparotomy. No ascites or succus was encountered upon entry via previous laparotomy. We safely divided fresh adhesions. The small bowel was closely examined from the terminal ileum to the ligament of Treitz. We found diffuse, mostly gaseous dilation of the entire small bowel, consistent with ileus, but no signs of ischemia or perforation. There were no transition points to suggest an early postoperative small bowel obstruction. Some segments of the jejunum had significant pneumatosis but were otherwise intact and well-perfused (Figure 4). The previous small bowel anastomosis was healthy and patent. We performed an incidental appendectomy. The large bowel also had no signs of necrosis or perforation. The abdomen was irrigated and closed in layers.

Her postoperative course was complicated by acute respiratory failure and hypotension requiring intubation, mechanical ventilation, and brief vasopressor support. The patient was subsequently weaned off the ventilator, downgraded, regained bowel function by postoperative day 4, and discharged back to the facility on day 10.

## Discussion

The pathophysiology of PI is not well understood, but suggested mechanisms include mechanical pressure tracking intraluminal gas into the intestinal wall via the mucosa, a change in the microbiota causing overgrowth of hydrogen-producing bacteria, or invasion and breach of the mucosal layer by gas-producing bacteria [1]. More recently, SARS-CoV-2 has been implicated as a possible cause of PI, suggesting its invasion of intestinal epithelial cells via ACE2 receptors as a possible cause [8].

Some suspect that the IL-6 inhibitor used to treat COVID-19 might be responsible for the PI cases seen in COVID-19 patients [1]. IL-6 inhibitor therapy has been implicated in intestinal perforation in patients with rheumatoid arthritis treated with IL-6 inhibitor [13]. Of the cases of PI in COVID-19 reported, many were treated with IL-6 inhibitors [9]. However, our patient was never treated with an IL-6 inhibitor but with an RNA replication inhibitor, molnupiravir. There have been no reported cases of PI with molnupiravir therapy in COVID-19 in the literature thus far, although symptoms of gastrointestinal distress with diarrhea and nausea have been reported [14]. It is unclear if this medication played any role in our patient’s findings.

Our patient had a benign intraoperative finding of an intact segment of the small bowel with PI without any signs of necrosis or ischemic insult, despite her concerning clinical presentation. Her ileus, caused by COVID-19 pneumonia, likely was the etiology of PI, as per mechanical PI theory. Of note, there was no PI on the CT scan during her previous admission for small bowel volvulus. The location of the current PI was not in proximity to the recent small bowel anastomosis. As discussed above, SARS-CoV-2 may invade the intestinal epithelium, leading to gastrointestinal manifestations, including abdominal pain, nausea, and PI. It may be suggested that our patient’s PI is secondary to the viral infection rather than the treatment modality or a thromboembolic event.

Benign PI has been seen in patients with obstructive pulmonary disease. The pulmonary theory of the development of PI explains this phenomenon. During a coughing spell, alveolar rupture may occur with gas dissecting through the mediastinum and then eventually making its way through the mesenteric root to the intestinal wall. PI in COVID-19 infection, with its upper respiratory symptoms, may also be secondary to this air tracking. Furthermore, patients with underlying pulmonary disease or chronic constipation with chronically increased intestinal intraluminal pressure may be at a higher risk of developing radiologic PI. Although the clinical significance of these findings may be variable, given the paucity of understanding regarding the association between COVID-19 infection and PI, further research is warranted.

## Conclusions

The pathophysiology of PI in patients with COVID-19 infection is not obvious and is typically associated with compromised bowel. Our case of PI in the setting of COVID-19 revealed benign operative findings, except for small bowel ileus, not uncommon in cases of pneumonia. The patient did not receive an antiviral agent potentially associated with PI in COVID-19. We consider this case of PI was secondary to the viral infection itself and ileus, rather than treatment modalities or a thromboembolic event. Further research is warranted to delineate the pathophysiology of PI in COVID-19.
